# From banks to burrows: Habitat preferences and nesting behaviours of platypuses in the Snowy River

**DOI:** 10.1002/ece3.70347

**Published:** 2024-12-24

**Authors:** Joseph Crane, Gilad Bino, Neil R. Jordan, Tahneal Hawke, Justine K. O'Brien

**Affiliations:** ^1^ Platypus Conservation Initiative, Centre for Ecosystem Science, School of Biological, Earth & Environmental Sciences University of New South Wales Sydney New South Wales Australia; ^2^ Taronga Institute of Science and Learning Taronga Conservation Society Australia Dubbo New South Wales Australia

**Keywords:** Australia, breeding, cryptic, egg yolk production, mammal, monotreme, nest, radio tracking

## Abstract

Platypuses are a unique freshwater mammal native to eastern Australia. They are semi‐aquatic, predominantly nocturnal, and nest in burrows dug into the banks of waterbodies. Quantifying nesting burrow characteristics is challenging due to the species' cryptic nature. We radio‐tagged 11 female platypuses during their breeding season (September to November) on the Snowy River, located their resting and nesting burrows by radiotracking, and assessed plasma triglyceride concentration as a biomarker of egg production. We quantified and tested for differences in height and distance from water of resting and nesting burrows, as well as for differences in both canopy and ground cover in the vicinity of resting and nesting burrows in comparison with background control sites in the area. Female platypuses displayed a strong selection for trees and shrubs, placing both their resting and nesting burrows within 5 m of these features. Compared with resting females, nesting females selected to dig nesting burrows higher above the river (nesting 1.98 m ± 0.27 SE vs. resting 1.15 m ± 0.10 SE) that were also further away from water (9.10 m ± 1.08 SE vs. 4.77 m ± 0.53 SE). Camera trap footage captured mice (*Mus musculus*) and black rats (*Rattus rattus*) entering two confirmed nesting tunnels on numerous occasions. During the first 3 weeks following the onset of nesting behaviour in two platypuses, rats entered the nesting tunnel a total of eight times and 31 times. Whether this is a previously unconsidered predator by invasive species remains to be evaluated. Synthesis: Riparian vegetation is a critical component of platypus habitat, providing stability for burrows, protection from predators, retaining high bank necessary to avoid inundation of burrows, and providing organic matter for nesting material and for abundant macroinvertebrate communities. Given ongoing declines and habitat degradation across their range, riparian habitat must be conserved and restored to promote breeding and population persistence.

## Graphical abstract

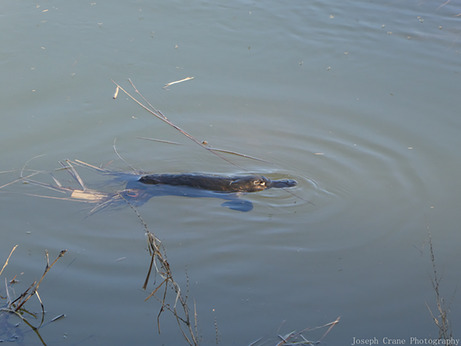

## 1 INTRODUCTION

Freshwater ecosystems support high biodiversity (Meyer et al., 1999) but are also experiencing unprecedented anthropogenically‐driven degradation (Grill et al., 2019). Continued mismanagement of freshwater ecosystems (Kingsford & Nevill, 2006) has placed numerous dependent freshwater species on a downward trajectory towards extinction. Particularly vulnerable are cryptic species, whose elusive nature complicates the task of gaining an accurate understanding of their habitat requirements (Clarke et al., 2003; Williams, 2016). Ensuring successful breeding, an essential aspect of long‐term species viability, thus becomes a considerable challenge. Among such species is the platypus (*Ornithorhynchus anatinus*), a globally unique and cryptic freshwater species. Platypuses are impacted by the range of human‐mediated threatening processes that affect freshwater systems and associated habitats more broadly (Grant & Temple‐Smith, 2003). Population declines have led to listing as ‘Near Threatened’ on the IUCN red list (Woinarsk & Burbidge, 2016), as well as a ‘Threatened’ status in Victoria (*Flora and Fauna Guarantee Act 1998*), and ‘Endangered’ in South Australia (*National Parks and Wildlife Act 1972*), given their disappearance from the state's mainland. Recent evidence suggesting declines across its range, coupled with our limited knowledge of its breeding behaviour and ecology (Bino et al., 2020; Hawke et al., 2020), underscore the urgency of improving quantification of habitat requirements for breeding.

The platypus is one of five living egg laying mammals of the Order Monotremata and the only living species of the *Ornithorhynchidae* Family (Grant & Fanning, 2007). Along with the Rakali (*Hydromys chrysogaster*), they are the only two semi‐aquatic mammals found in Australia's freshwater systems (Grant & Temple‐Smith, 2003). The platypus is distributed across eastern mainland Australia (Queensland, New South Wales, Victoria, Australian Capital Territory), Tasmania, and King Island (Grant & Fanning, 2007), as well as an introduced population on Kangaroo Island (Bino et al., 2019). Within this range, they occupy both lotic and lentic habitats (Serena et al., 2001), feeding exclusively in the water (Serena et al., 1998) on a range of benthic invertebrates along stream edges, pools, and riffle habitats (McLachlan‐Troup et al., 2010). During the breeding season (late winter to early spring), platypuses also conduct courtship and mating in the water (Thomas, Parrott, et al., 2018). Whilst feeding and mating is conducted in the water, platypuses use the banks and construct somewhat ephemeral resting and more long‐term nesting burrows (Serena, 1994).

Following courtship and mating, while embryogenesis is assumed to occur, females platypus dig a complex nesting burrow in river and creek banks (Thomas, Handasyde, et al., 2018). In the wild, nesting burrows have been found to range from 4.3 m to 9.3 m in length (Temple‐Smith, 1973), with some entrances found to be 65–80 cm above the water (Serena, 1994) and 1.8 m–2.6 m away from the water (Temple‐Smith, 1973). Due to the cryptic nature of the platypus, these data are derived from only a few nesting burrows across different localities and waterways. They are dug into steep, consolidated banks (Temple‐Smith, 1973), in contrast to resting burrows, which tend to be 1–2 m from the water and entrances tend to be underwater or at the water surface (Serena, 1994). Nesting burrows can be complex structures, with multiple tunnels and up to three separate entrances (Thomas, Handasyde, et al., 2018). Burrow chambers are known to have a maximum height of 20 cm and width of 28 cm, located 23–30 cm below the ground surface (Temple‐Smith, 1973). Platypus females are known to block up (“pug”) sections of the tunnel, potentially to protect from predators or keep conditions at an optimum for nesting, preventing desiccation of eggs and young (Burrell, 1927; Thomas, Handasyde, et al., 2018).

Platypuses have a relatively slow recruitment rate, with only half of the females breeding in any given year (Bino et al., 2015). Knowledge of platypus rearing of young is predominantly derived from managed breeding efforts. Following mating, zoo‐based females spend approximately 8 h a day over 2–5 nights collecting vegetation from the water's surface for their nests, using their tail to drag it into the burrow (Thomas, Handasyde, et al., 2018). Females then forage for 3–8 days before retiring to the burrow to lay 1–3 eggs (Hawkins & Battaglia, 2009; Thomas, Handasyde, et al., 2018), which are incubated for approximately 10 days (Griffiths, 1978). During the first 15 days after laying eggs, females only leave the burrow for a total of 7–8 h to feed (Thomas et al., 2019), compared with a non‐breeding platypus which forages for approximately 11 h per day (Bethge et al., 2003). In ex situ breeding programs, females are known to lactate for approximately 4 months (Thomas et al. 2019) until the young leave the nest. The energy costs of lactation are considerable, with the dietary caloric intake in the last month of lactation double that of a non‐lactating female (Thomas et al. 2019). Platypus young are dependent on their mother until they leave the nest, during this time they are vulnerable to flooding, disease, and predation, making decision on nest location crucial.

Zoo‐based breeding programs have had limited success to date. Since the first captive bred platypus in 1943, breeding has only been successfully facilitated in six pairs, some of which were successful on a number of occasions (Thomas, Parrott, et al., 2018, Thomas September 2022 pers. comm.). Limited success has been attributed to deficiencies in knowledge of breeding requirements. Recently, key features of nesting burrows and nest vegetation material in zoo‐based platypuses have been identified (Thomas, Handasyde, et al., 2018). However, burrow characteristics and positions have only been recorded in a small number of localities across the species' distribution. For example, Serena (1994) documented habitat attributes for three nesting burrows, but this aspect was not the study's primary focus. More information on the habitats that wild female platypuses prefer for their nesting burrows is urgently needed, for informing husbandry management of ex situ assurance populations, and for in situ conservation efforts.

In this study, we assessed biotic and abiotic characteristics of platypus burrows on the Snowy River, New South Wales. We tagged and radio‐tracked 10 female platypuses between 20 September and 28 November 2021, coinciding with their breeding period as indicated by behavioural and physiological markers. We identified both resting and nesting burrows and evaluated habitat preferences against available habitat. We examined several aspects, including distance from the water, height above water, and proportions of canopy and ground cover. Based on the current knowledge, we hypothesised that female platypuses would choose to build their nesting burrows higher above the water and further away from the water than more ephemerally used resting burrows to avoid risk of inundations. We hypothesised that female platypuses would select to build their nesting burrows in the vicinity of shrubs and trees which are able to support the stability of the nesting chamber.

## 2 METHODS

### 2.1 Study area

This study focused on a 3.64 km river section of the Snowy River in New South Wales, Australia, with an elevation of 760 m, approximately 6 km upriver of the Dalgety township and 18 km downriver of Jindabyne Dam. This area was selected following previous research indicating large numbers of platypuses (Hawke, Bino, Kingsford, Iervasi, et al., 2021). River flows for the Snowy River are regulated and throughout the year schedules several large releases of water with daily peak flows over 4000 ML/day (flushing flows). Over the study period, three flushing flow releases were performed (6 October, 19 October, and 9 November 2021) with peaks of 10,362 ML/day (119.9 m^3^/s), 4699 ML/day (54.4 m^3^/s), 4263 ML/day (49.3 m^3^/s) respectively, and an additional natural increase in flow volumes from local rainfall. During the study period, daily flows ranged between 380 ML/day to 10, 362 ML/day.

**FIGURE 1:**
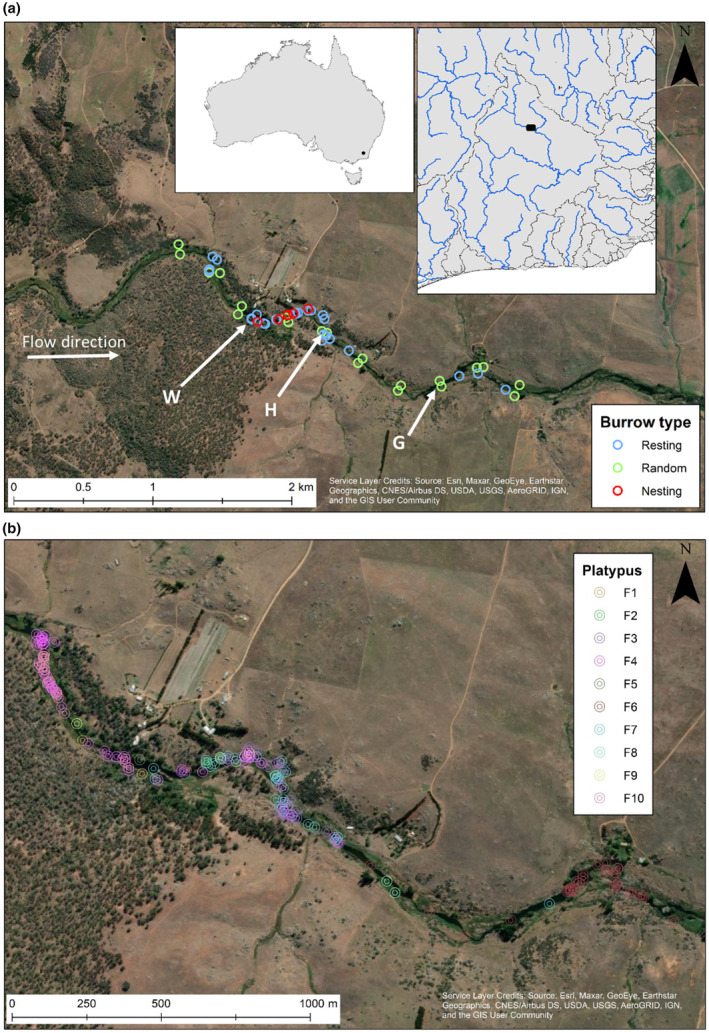
(a) Nesting burrow (red circles), resting burrow (blue circles), and random sites (green circles), along with the three pools surveyed (W, G, H) within the study area (~3.64 km) along the Snowy River, Australia (two insets with black marks showing the study area); and (b) locations of the 10 tagged female platypuses during the tracking period 13/9/2021–25/11/2021.

Trees in the study site consisted of predominantly *Eucalyptus pauciflora*, *E. viminalis*, *E. dalrympleana*, *E. stellulata*, *Populus* spp. and sparse *Salix* spp. Shrubs along the banks consisted predominantly of *Leptospermum* spp., *Rubus fruticosus* and *Mirbelia oxylobioides*. Considerable cattle presence was observed on the northern bank (Figure 1) commencing from the farthest downriver nesting burrow continuing downriver for 1.6 km. Although there was less livestock along the southern bank, there were frequent sightings of deer. Potential platypus predators including cats, domestic dogs, and foxes were all sighted in the study area.

Trapping of platypuses was undertaken on two separate occasions, comprising a total of 10 nights. The first was 10–15 September 2021 and the second 12–15 October 2021, coinciding with the onset of the breeding season in late winter or early spring (Grant et al., 2004). Previous research on the Snowy River indicated emergence of juvenile platypuses in February, suggesting the breeding season in this region was late September to early October (Hawke, Bino, Kingsford, Iervasi, et al., 2021), similar to that on the Shoalhaven River (Grant et al., 2004). Three sites were selected along the river, based on the previous captures (Hawke, Bino, Kingsford, Iervasi, et al., 2021) and referred to as the ‘W', ‘G' and ‘H' pools hereafter.

### 2.2 Platypus trapping

To catch platypuses, we used unweighted mesh nets (80 mm multifilament nets 50 m × 2 m), (Bino et al., 2018), which were set from 16:00 h. to 00:00 h. using a small 6 ft. punt. Nets were visually checked every few minutes with a spotlight, immediately removing platypuses. We also physically examined nets every hour to ensure no snags were weighing down the nets. Platypuses were retrieved from the nets and placed in pillowcase in a quiet location until processed. Platypuses were assessed for injuries to ensure they are in a suitable condition to be anaesthetised. Once deemed suitable, they were anaesthetised within an induction chamber and then with a face mask connected to a vaporiser delivering isoflurane (Pharmachem, 5%) in oxygen (1–3 L/min) (Chinnadurai et al., 2016; Fiorello et al., 2016; Vogelnest & Woods, 2008). Body temperature, heart rate, and blood oxygen were monitored continuously throughout processing using an oximeter Darvall H100N (Bino et al., 2018). Once anaesthetised, platypuses were weighed, measured, and assessed for sex and using spur morphology age (Serena, 1994; Williams et al., 2012). Platypuses were microchipped with a Passive Integrated Transponder Tag (Trovan) (Bino et al., 2021) or scanned to determine if it had been previously captured.

### 2.3 Estimating female platypus reproductive cycle stage

Blood samples were collected for general health monitoring and to determine plasma concentrations of triglycerides, an egg yolk precursor index in other egg laying species (O'Brien et al., 2016). Blood (2 mL) was collected from the bill sinus (Whittington & Grant, 1983) into heparinized (lithium) tubes and remained at ambient temperature for up to 6 h before being transferred to 5°C–10°C. Samples were centrifuged the following morning for 10 min at 1000*g* and the plasma stored at −80°C until biochemistry analysis. Frozen–thawed plasma was analysed for triglyceride concentration (mmol/L) without dilution in 2022 by Vetnostics Laboratory, using a Cobas 8000 modular analyser (Roche Diagnostic Systems). Baseline concentration of triglyceride was designated as 0.67 mmol/L, which reflects mean concentrations observed for female platypuses in New South Wales during December to May (Stewart et al., 2021). This period represents the post‐lay stage when ovarian activity is absent or minimal.

### 2.4 Radio tagging and tracking

Adult females were fitted with radio transmitters (Lotek MST‐930 VHF) with a built‐in motion sensor to indicate lack of movement for periods greater than 6 h, and an operating battery life of ~113 days. Transmitters were fitted by initially shaving a small (2 × 2 cm) patch of fur anterior to the tail on the platypus's dorsal side, and then glued using a fast‐setting non‐toxic superglue (Thomas et al., 2019). Platypuses were released in the same pool they were captured after removal of nets from the water and after being held for up to 6 h. Radio transmitters have not been observed to interfere with mating behaviour in platypuses (Thomas, Handasyde, et al. 2018).

Platypuses were radio tracked a minimum of once per day between 20 September – 31 October and opportunistically between 13 and 19 September, and 1 and 28 November. Most days platypuses were tracked twice, once in the morning as early as 05:30, and again between midday and midnight. In instances where platypuses were thought to be nesting (see below), tracking efforts were increased (maximum of 12 locations per day). Radio tracking was undertaken by foot using a Lotek SRX1200 receiver and a handheld three‐element Yagi antenna (Kenward, 2000). Platypus locations were determined and recorded by tracking the animal until it was directly observed in the water or confirmed within a burrow identified either directly above the burrow or triangulating to the opposite bank when crossing the river was not possible. For platypuses tracked to burrows, GPS coordinates were recorded at the point where the strongest signal was detected, to within a 1 m radius. Transmitters were assumed to have fallen off if they emitted an inactivity signal for over a week.

### 2.5 Identifying nesting burrows

We identified nesting burrows if a female platypus met three key criteria: (1) The female consistently used the same burrow for at least seven consecutive days. Although egg incubation is estimated to take 10 days, and females typically leave their young for periods greater than 24 h only after 44–52 days of maternal care (Thomas et al., 2019), the shorter seven‐day period was deliberately chosen to also capture cases of failed breeding attempts. Studies of zoo‐based platypuses have documented instances where nesting females abandoned nests after 2, 10, 11, and 18 days following egg laying (Thomas, Handasyde, et al., 2018). (2) The female exited the burrow no more than three times during the seven‐day period. Zoo‐based nesting females initially remain inside the nesting chamber for more than 80 h (±18 SE) (Hawkins & Battaglia, 2009) or approximately 4 days (Holland & Jackson, 2002), and are believed to lay eggs shortly after retiring to the nest (Thomas, Parrott, et al., 2018). The subsequent 2–3 foraging trips typically occur after an average interval of 43 h (±6 SE), followed by an extended burrow stay of 65 h (±10 SE) (Hawkins & Battaglia, 2009). In captivity, females tend to leave the burrow every few days to feed for an average of 82 min (±11 SE) during the first 15 days after entering the nesting burrow (Thomas et al. 2019). (3) The transmitter detected some level of activity within the burrow during the seven‐day period, and the burrow was situated at least 1 m from the water's edge, to rule out instances where transmitters may have detached at burrow entrances or in the water. This approach is grounded in documented nesting behaviour patterns, where females exhibit continuous occupancy during egg incubation and early rearing periods. While this method may not account for every nuance, it aligns closely with established platypus behaviour patterns and provides a robust framework for accurately identifying nesting burrows.

We set 15 camera traps (Reconyx Hyperfire 2) in front of potential burrow entrances to confirm use, detect female platypuses carrying nesting material, and provide a measure of frequency of burrow use. Camera traps were installed at distances 0.15–2 m from burrow entrances. Distances and angles were based on the burrow morphology. The cameras were configured with high trigger sensitivity with motion video at 720 P, with a 5‐second delay between video captures. The cameras were also set to “No‐Glow” infrared mode to minimise disturbance during nocturnal activity, with video lengths set to 10 s. Camera trap footage from 17 September – 26 October was reviewed every 1–3 days during the tracking period and 27 October – 29 November after detection of nesting females had concluded. When possible, platypuses were recorded carrying nesting material in the water using a hand‐held camera (Panasonic Lumix DMC TZ80) and identified using radio tracking.

### 2.6 Breeding timeline

For the females observed carrying nesting materials, we estimated the date of mating by subtracting 7–15 days, which represents the reported duration between the last day of mating and the first day of nest‐building, as well as subtracting known durations of nest building (2–5 days, inclusive of day of observation) for successfully reproducing females (Hawkins & Battaglia, 2009; Thomas, Handasyde, et al., 2018). In other words, 8 to 19 days were subtracted from the date of observed nest‐building to estimate the date of mating. The estimated date of retirement to the nesting burrow was determined by adding the reported range this event occurs following the last day of nest building for successfully reproducing females (2–5 days), (Hawkins & Battaglia, 2009) along with the known durations of nest building. This meant that 3 to 9 days were added to the date of observed nest‐building to estimate date of retirement to the nesting burrow for impending egg lay (i.e. for those females where nest‐building was observed on only one date). The date ranges were also checked for alignment with tracking data indicating a female spent at least seven consecutive days in the nesting burrow. For females not observed to exhibit nest‐building behaviour, estimated dates of mating were less definitive and based on the tracking data, subtracting 13–23 days from the first date a female then spent at least seven consecutive days in the nest burrow (Thomas et al., 2019).

### 2.7 Control sites

To determine whether nesting or resting burrows were preferentially situated in particular habitats relative to their availability, we created control sites for comparison. Once all nesting and resting burrows were determined (by 31 October 2021), upper and lower limits for the study site were defined. This was done by measuring 500 m upriver (upper point) from the farthest upriver resting burrow and measuring 500 m downriver (lower point) from the farthest downriver burrow using Google Earth (Google Earth, 2022). Daily tracking in this study indicated that females remained within this region and did not exceed the selected upper and lower points. The distance between the upper point and lower point were measured along the middle of the river, equating a total distance of 3.64 km. This river section was then equally divided into 10 sections (364 m each), with a midpoint placed in the middle of each section (*n* = 10). Twenty points were then selected, 10 on either side of the riverbank, in line with the midpoint. This was done to reduce the chances of biased selection of habitat areas on the map and in the field. Coordinates were extracted (to 7 decimal places) and located in the field. Once locations were identified, the closest point to the river was marked and then a final control site was determined at 9.10 m from the water's edge, representing the mean distance of all identified nesting burrows. As water height was influenced by water releases from Jindabyne Dam, sites were only marked on days when the daily flows were between 513 ML/day – 568 ML/day (https://www.snowyhydro.com.au). We acknowledge that control sites may have overlapped with undocumented burrows any may have introduced false negatives but we consider these to be negligible given the large number of females tagged in the area which lies in the higher end of estimated densities in the area (Hawke, Bino, Kingsford, Iervasi, et al., 2021).

### 2.8 Habitat assessments

We undertook habitat assessments for all six nesting and 10 resting burrows as well as control sites between 31 October and 29 November 2021. For each nesting and resting burrow, we measured the true distance along the ground to the water, the horizontal distance to the water's edge, as well as the height above the water line. To minimise the influence of river height on measurements, the assessments were carried out on days when releases from Jindabyne Dam were between 513 and 607 ML/day.

We assessed canopy and ground cover over five transects originating from above the burrow location. One ‘water transect’ (W) commenced at the top of the burrow and continued to the water's edge. The other four transects, originated from the burrow and extended 5 m outwards, the first at a 45° angle to the river, and the rest each 90° apart, numbered clockwise (1, 2, 3, 4). Along each transect, the dominant ground cover feature was assessed at 10 cm increments and classified as Grass, Shrub, Tree, Log/Rock, Bare, Water and tallied to the proportion for every 1 m. Trees and shrubs were classed based on Specht's vegetation system (Specht, 1970), with reference to Costermans (Costermans, 1981). Trees were classified as such if they were taller than 5 m, usually with a single stem, while shrubs were shorter than 8 m and frequently had many stems arising at or near the base. Trees and shrubs were identified to Family level, and when possible, to a species level using Native Trees and Shrubs of South‐Eastern Australia (Costermans, 1981), to ensure they had been classified correctly as a tree or shrub. We then summed the total length of each dominant feature along with of the five transects and calculated their respective proportions. Canopy cover was estimated by looking up perpendicularly with one eye looking through a hand replicating a sighting tube. This was done at every 1 m interval along the transect and estimating the proportion of cover directly above.

We focused on canopy cover because of its assumed critical importance in providing suitable microhabitat conditions for nesting and resting sites. Canopy cover from trees and shrubs plays a vital role in maintaining soil stability, which is essential for the integrity and longevity of burrow structures. High canopy cover contributes to the microhabitat by retaining soil moisture (Keppel et al., 2017). Furthermore, the presence of trees and shrubs improves soil stability, reducing erosion and maintaining the structure needed for burrow excavation (Zuazo & Pleguezuelo, 2009). In addition, we explored ground cover selection to assess its importance for predator avoidance, its possible effects on microhabitat conditions, and providing necessary materials for nest building.

### 2.9 Statistical analysis

We evaluated differences between nesting and resting burrows in height above water, horizontal distance to the water, and distance to the water along the ground. We used the Wilcoxon Rank Sum Test. This non‐parametric test is suitable for comparing two independent groups when the data do not necessarily follow a normal distribution. The test was executed using the wilcox.test function available in the R statistical software (R Development Core Team, 2023).

We tested for differences in canopy cover between nesting burrows, resting burrows, and control sites. We did this for the first 3 m along the Water Transect measured from the water's edge towards, and above the burrow's entrance. Similarly, we test for differences across the five, 5 m transects (Water, 1, 2, 3, 4) directly above the burrow. We also tested for differences in shrub and trees ground cover of along the Water Transect measured from the water's edge towards, and above the burrow's entrance.

As canopy and ground cover were both continuous variable restricted to the unit interval of 0–1, we used a maximum likelihood regression approach for beta‐distributed dependant variables (i.e., continuous using the *‘betareg’* package (Zeileis et al., 2021). We transformed proportional cover using:

$$\frac{\left(y∙\left(n-1\right)+0.5\right)}{n},$$

where *n* is the sample size (Smithson & Verkuilen, 2006), as recommended in the ‘*betareg*’ package (Zeileis et al., 2021). All three models can be represented as:

(1) $$\text{logit}\left(y_{i}\right)=\beta_{0}+\beta_{1}\cdot \;{\mathrm{CRN}}_{\text{rest}}+\beta_{2}\;\cdot {\mathrm{CRN}}_{\text{control}}+\epsilon_{i}$$

where *y*_i_ is the response variable for the i‐th observation, *β*_0_ is the intercept, *β*_1_ is the coefficient for rest burrows, *β*_2_ for control sites, and $\epsilon_{i}$ is the error term, with nest burrows as the reference category. Model validation was performed by examining the residuals and ensuring that they were randomly distributed, indicating a good fit. We checked for patterns in the residuals against fitted values and predictors to verify the assumptions of the beta regression model. Additionally, diagnostic plots were created to assess the goodness of fit and identify any potential issues with the model. We used the *‘emmeans’* package (Lenth, 2022) to calculate the estimated marginal means of the model for interpretation of effects.

We also assessed the preference for ground cover by platypuses by using the Manly's Selective Index (Manly et al., 2007). This approach tests for differences between the proportion of available habitat to that selected by each animal. We defined control sites which were randomly sampled as available habitat. We used the ‘*widesII*’ function to calculate selection ratios where availability of resources is the same for all animals while use is measured for each animal, using the ‘*adehabitatHS’* package (Calenge, 2020). The Manly selection ratio for use and availability of each ground cover class was calculated as:

$$w_{j}\frac{u_{j}}{a_{j}}$$

where *u*_j_ is the proporton of use of the ground cover class *j* and *a*_j_ is the proportion of availability of this ground cover class. Habitat selection ratios were significant if their 95% confidence intervals did not include 1; values >1 indicated selection while values <1 indicated avoidance (Manly et al., 2007). By using Design II, statistical inferences treat each animal as a replicate (Manly et al., 2007). With Design II, log‐likelihood test statistics are calculated to test identical use of habitat by all animals (χ^2^L1), test overall habitat selection as a group (χ^2^L2), and test if animals are on average using resources in proportion to availability, irrespective of whether they are selecting the same or not (*χ* ^2^L2 − *χ* ^2^L1) (Manly et al., 2007). The true available proportions were unknown (Manly et al., 2007).

## 3 RESULTS

### 3.1 Trapping

A total of 17 platypuses were captured over the 10 nights, consisting of 11 adult females (883 g ± 86 SD, 770–1000 g) and six males (1280 g ± 180 SD, 1140–1600 g) (Figure 1 and Appendix S1), representing a minimum capture density of 4.67 platypuses per km (Appendix S2). Only two females caught during this study were recaptured during the 2021 trapping period. Three adult females were recaptured from surveys undertaken in 2017, determined at the time to be adults.

### 3.2 Tracking

Eleven platypuses were tracked over a total of 72 days (Table 1). One female (F11) was not detected again after release even though tracking attempts were made at least once a day from 18 October – 20 November 2021. Return tracking sessions ranged from 35 min (1.3 km) to 5 h 5 min (8.8 km) depending on locations of platypuses and difficulty in pinpointing location. The number of days that each individual platypus was located varied from 7 days to 70 days, dependant on possible tag dislodgment (Table 1). Platypus movement ranges were positively associated with number of days tracked (*r* = .48), ranging between 0.223 km (F1) to 2.08 km (F6), averaging 0.814 km ± 0.693 SD (Figure 1, Appendix S3). Platypuses were not located outside of the study area of 3.64 km during the time tracking was conducted.

**TABLE 1:** Tracking period of female platypuses, total distance (range) along river covered [m], number of days and times located, estimated dates of mating, observed nesting behaviour, estimated date of retirement to nesting burrow, and plasma concentrations of triglycerides at time of tagging.

Platypus	Start–end (2021)	Range [m]	No. days/times located	Estimated mating	Observed nest‐building behaviour	Estimated retirement to nest burrow	Plasma triglycerides (mmol/L)
F1	11 Sep–25 Nov	223	70/161	6 Sep (TD)		20 Sep (TD)	1.5 (10 Sep)
F2	16 Sep–5 Oct	497	18/60	4–15 Sep	23 Sep	26 Sep‐2 Oct	2.9 (11 Sep)
F3	13 Sep–10 Nov	829	50/121	24 Aug–3 Sep (TD)		7‐17 Sep (TD)	0.3 (11 Spe)
F4	19 Sep–25 Nov	1440	63/142				1.7 (16 Sep)
F5	19–26 Sep	44	8/20	1–12 Sep	20 Sep	23–29 Sep	2.8 (16 Sep)
F6	21 Sep–23 Nov	2083	46/95	4–14 Sep (TD)		18–28 Sep (TD)	1.8 (17 Sep)
F7	19 Sep–23 Oct	1627	35/109	4–14 Sep (TD)		18–28 Sep (TD)	2.6 (17 Sep)
F8	20 Sep–25 Nov	801	63/158	6–17 Sep	25 Sep	28 Sep–4 Oct	7.8 (17 Sep)
F9	15–22 Oct	79	7/11				4.0 (17 Oct)
F10	15 Oct–18 Nov	504	34/60				1.8 (15 Oct)
F11	15 Oct–20 Nov						2.5 (15 Oct)
Untagged (observed)				6–16 Sep	23–25 Sep	26–30 Sep	
Untagged (captured)							7.5 (17 Oct)

*Note*: Estimated dates were based on either Tracking Data (TD) or observed nest‐building behaviour.

### 3.3 Nesting behaviour

Four female platypuses (F2, F5, F8 and untagged female) were documented carrying nesting material on the 23 September, 20 September, 25 September, and 23 September, respectively. The three tagged platypuses had plasma triglyceride concentrations in mid‐September well above baselines at 2.9, 2.8, and 7.8 mmol/L, respectively, indicating active egg yolk production. An untagged female platypus caught at Dalgety, New South Wales (approximately 8 km downstream of the study area) had a very high triglyceride concentration of 7.5 mmol/L in mid‐October (Table 1). Four additional females (F1, F3, F6, and F7) met the criteria for breeding based on the radio‐tracking data, although plasma triglyceride concentrations were somewhat lower at 1.5, 0.3, 1.8, and 2.6 mmol/L, respectively. Of the seven tagged platypuses, six possible nesting burrows were located (F1‐F3, F6‐F8). All nesting burrows were located along a single 405 m section (11.5%) of the study site (Figure 1). F3 had below baseline plasma triglyceride at time of tagging but exhibited nesting behaviour as soon as tracking commenced, utilising the same burrow for 14 consecutive days, suggestive of egg laying prior to tagging. Both F3 and F7 were considered to have abandoned their nesting burrows, with both not located again in their burrows after the 29 September and 4 October, respectively. F9 may have commenced nesting in burrow (F9B2) on the 19 October, given the distance of the burrow from the river (4.25 m, at a release of 3871 ML/day), however, the tag became inactive\fell before nesting behaviour was confirmed. F6 abandoned her nesting burrow (F6B1) following a significant rise in water levels coinciding with a large water release from Jindabyne Dam on the 6 October. At 14:32 h. on the 6 October, burrow F6B1 was 1.40 m from the water's edge and 0.22 m above the water (from the topsoil) (Table 2). Water levels continued to rise after measurements were taken. Measurements from the highest watermark placed the water within 0.35 m of the burrow's midpoint and an estimated 0.07 m above water. F4 was not considered to have been breeding as she was located four times outside of her burrow within a 7‐day period. However, this criterion was developed based on a small number of zoo‐based female platypuses which may exhibit different behaviours to those by wild platypuses. Of all the tracked females, successful breeding was likely for only F8, who remained in the same burrow with an active signal from 20 September until the 25 November. F1 and F2 may have also successfully bred, but tag inactivity, suggestive of tag dislodgement prevented confirmation.

**TABLE 2:** Distances (ground, horizontal) and height of the nest chamber to the water at varying releases from Jindabyne Dam, measured from the ground above located burrow chamber.

	Ground distance to water [m]				Horizontal distance to water [m]				Height above water [m]			
Dam daily peak release (ML/day)	10,362	4699	2173	607–557	10,362	4699	2173	607–557	10,362	4699	2173	607–557
F1B1	2.01	–	6.25	7.80	1.95	–	5.80	7.12	0.31	–	1.05	1.59
F2B1	2.65	3.72	4.35	6.39	2.35	3.19	3.80	5.55	0.62	1.07	1.09	1.87
F3B2	7.70	–	9.65	13.37	7.11	–	8.70	12.28	2.20	–	3.02	3.14
F6B1	1.40	2.77	4.80	6.75	1.32	2.61	4.50	6.01	0.22	0.62	0.95	1.34
F7B2	5.65	6.79	8.20	10.30	5.15	6.16	7.65	9.48	1.30	1.63	1.85	2.30
F8B1	3.45	6.20	7.60	9.96	3.00	5.93	6.90	9.49	1.08	1.22	1.34	1.67

### 3.4 Burrow use

**FIGURE 2:**
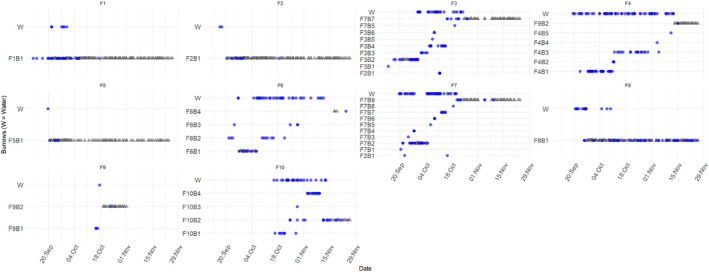
Locations (W‐water or burrow code) of tracked platypuses between 13/9/2021 and 25/11/2021 by activity signal (Active – purple circles, Inactive – grey triangles).

A total of 29 resting burrows and six nesting burrows were located, with a mean 3.8 burrows per platypus and a range of 1–9. Four burrows (1 nest and 3 rest) were found to be used by multiple platypuses but never at the same time (Table 1 and Figure 2). F3 used a burrow (19 October) after F7 (6 October, F7B5, resting burrow), F4 used a burrow (13 November) after F9 (19 October, F9B2, resting burrow), and F3 used another burrow (15 October) after F7 (12–14 October, F7B7, resting burrow). F3 was also located within 1 m of F2's suspected nesting burrow (F2B1) (11 October), albeit the signal suggested she was moving, so it is uncertain whether she was in a tunnel or in the burrow chamber. However, this suggests that F2 may have abandoned the burrow after nesting (observed 23 September). The burrow F7B5 was only used on two occasions (once by F7 and once by F3), coinciding with two large flushing flow releases from the dam on the 6 October and 19 October, when flows peaked at 10,362 ML/day and 4699 ML/day, respectively. This burrow was in a side flow of the river and in lower flows the river was over 30 m away from this burrow, whereas in a high flow of 4699 ML, distance from top of burrow chamber to water was 4.51 m. All nesting burrows were located on the banks of pools, characterised by slow moving water. Multiple video captures were made in two separate nesting burrow tunnels. These showed F8 and an untagged platypus arriving but not leaving, suggesting multiple entrances to two separate nesting burrows.

Video footage of platypuses entering burrows and using tunnels were captured at a total of six separate burrows, two of these were confirmed nesting burrows. One burrow entrance was approximately 8 m from the water's edge, this entrance was unconfirmed whether it was for a nesting or resting burrow, although a nesting burrow had been located 5 m further up the bank (F3B2). The two confirmed nesting burrow entrances were both underwater, footage was captured from an open section of the tunnel close to the water's edge. One nesting burrow tunnel was confirmed to be associated with a tagged female, F8, and the other was an untagged female (UF). This female was confirmed to be nesting as footage revealed UF carrying nesting material through the tunnel on numerous occasions. Footage of F8's nesting burrow tunnel revealed at least another two platypuses using the same entry from the river. Visual inspection of the burrow entrance revealed an initial tunnel underwater accessing the river before splitting into two separate tunnels. In a separate location, footage of the nesting tunnel associated with UF showed at least two different platypuses entering the tunnel, including a tagged female. In each nesting tunnel, footage revealed non‐nesting platypuses entering part of the tunnel before promptly turning around and exiting, not progressing to the nesting burrow chamber.

Camera trap footage also revealed a cat (*Felis catus*) on two separate occasions passing the tunnel entrance to a platypus nesting burrow (F3B2), as well as a domestic dog (*Canis familiaris*) which began digging up an exposed section of the tunnel for the nesting untagged female (UF). Mice (*Mus musculus*) and black rats (*Rattus rattus*) entered two confirmed nesting tunnels numerous times. During the first 3 weeks of when each female platypus commenced nesting (UF and F8), rats entered the nesting tunnel a total of 8 times (range 0–2 times a day) for UF and 31 times (range 0–4 per day) for F8.

Measurements of two confirmed burrow entrances were (1) 77 mm wide and 89 mm high and (2) 92 mm wide and 54 mm high. An exposed platypus nesting tunnel (open topside by 30 cm) was also opportunistically measured, with end closest to the water measuring 140 mm wide and 90 mm high, the other closer to the nesting burrow 120 mm wide and 50 mm high.

### 3.5 Habitat preferences

**FIGURE 3:**
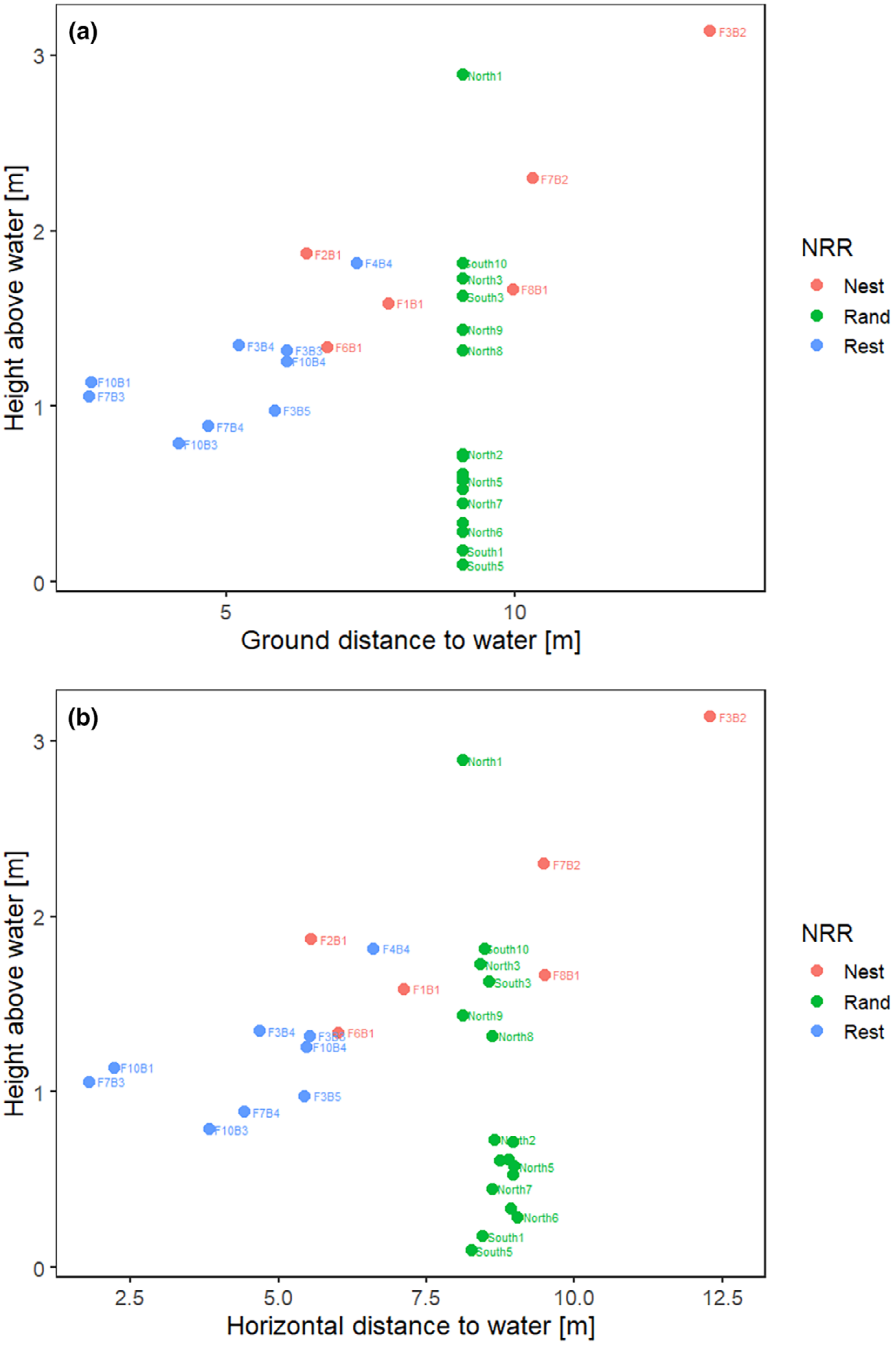
Scatter plots between (a) burrow height and distance to water along the ground and (b) burrow height and horizontal distance to water (nesting burrow = red, resting burrow = blue, random = green).

Nesting burrows were significantly further away from the water's edge (9.10 m ± 1.08 SE) compared with resting burrows (4.77 m ± 0.53 SE; Wilcoxon rank sum, *p* = .003, Table 2, Figure 3). Similarly, the horizontal distances of nesting burrows were significantly further from the water's edge (8.32 m ± 1.05 SE) compared with resting burrows (4.28 m ± 0.50 SE, *p* < .001). Nesting burrows were also significantly higher above water (1.98 m ± 0.27 SE), than were resting (1.15 m ± 0.10 SE; *p* = .003).

**FIGURE 4:**
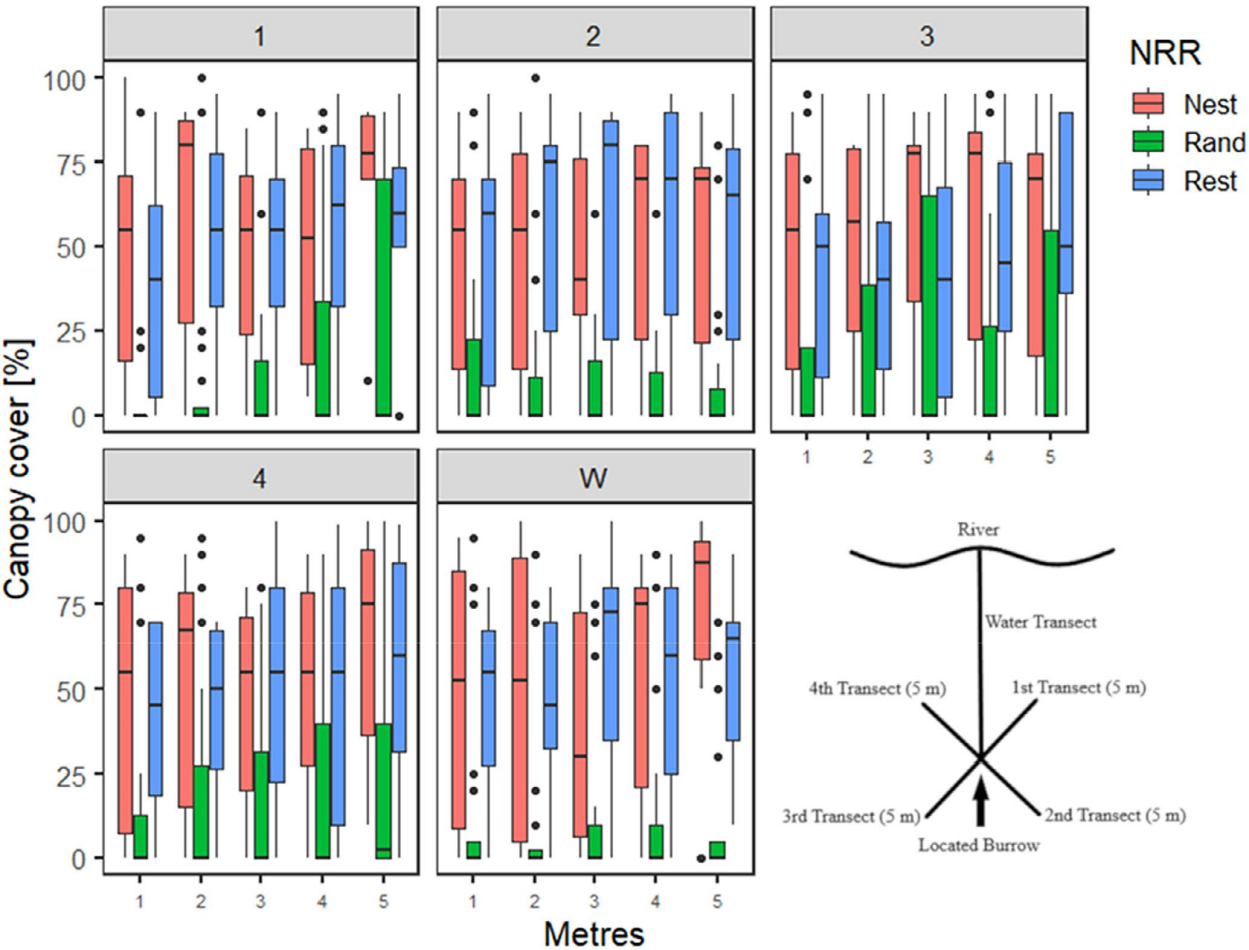
Canopy cover for each transect (1,2,3,4, W) at 1 m intervals above nesting, resting and random sites.

Proportion of canopy cover within 5 m above burrows was significantly greater above nesting (0.44 ± 0.03 SE, *p* < .001) and resting (0.41 ± 0.02 SE, *p* < .001) in comparison with that above control burrows (0.19 ± 0.01 SE, Pseudo *R* ^2^ = .23, Table 3, Figure 4). No significant differences were detected between nesting and resting burrows in this regard (*p* = .514). Canopy cover above burrow entrances (3 m from the water's edge), was significantly greater above nesting (0.44 ± 0.06 SE, *p* < .001) and resting (0.54 ± 0.05 SE, *p* < .001) compared with control sites (0.14 ± 0.02 SE, *p* < .001), but did not differ between nesting and resting (*p* = .184, Pseudo *R* ^2^ = .56, Table 3, Figure 4). The proportion of shrub‐tree presence within 3 m of the water was greater in nesting burrows (0.28 ± 0.06 SE, *p* = .046) in comparison with control sites (0.16 ± 0.03 SE), but not between resting (0.23 ± 0.04 SE, *p* = .115) and control sites, nor between nesting and resting burrows (*p* = .431, Pseudo *R* ^2^ = 0.17, Table 3, Figure 4). Five of the six nesting burrows were not on active farming land with frequent livestock presence (cattle and horses), although they were subject to infrequent feral deer and horse activity. The only nesting burrow (F3B2) that was on farmland was under a large tree and 5 m away from non‐farming land. All six nesting burrows had at least one wombat burrow within 5 m, seven of the 10 resting burrows had at least one wombat burrow within 5 m, and only 40% (8/20) of the control sites had at least one wombat burrow within 5 m.

**TABLE 3:** Summary statistics of a Generalised Linear Model of effect of burrow type (nesting\resting) on canopy cover above burrow chamber, canopy cover above burrow entrance (water transect), and shrub presence near burrow entrance.

Parameter	Estimate	SD	*z* Value	*p*r(>|z|)
Canopy cover over burrow chamber – 5 × 5 m transects				
Intercept	−0.259	0.105	−2.47	.013
Rand	−1.199	0.120	−10.02	<.001
Rest	−0.087	0.133	−0.65	.514
Canopy cover water to burrow entrance – water transect 1 × 3 m				
Intercept	−0.230	0.241	−0.946	.344
Rand	−1.633	0.281	−5.815	<.001
Rest	0.403	0.305	1.319	.187
Shrub presence water to burrow entrance – water transect 1 × 3 m				
Intercept	−0.946	0.293	−3.226	.001
Rand	−0.718	0.320	−2.241	.025
Rest	−0.285	0.355	−0.803	.422

**FIGURE 5:**
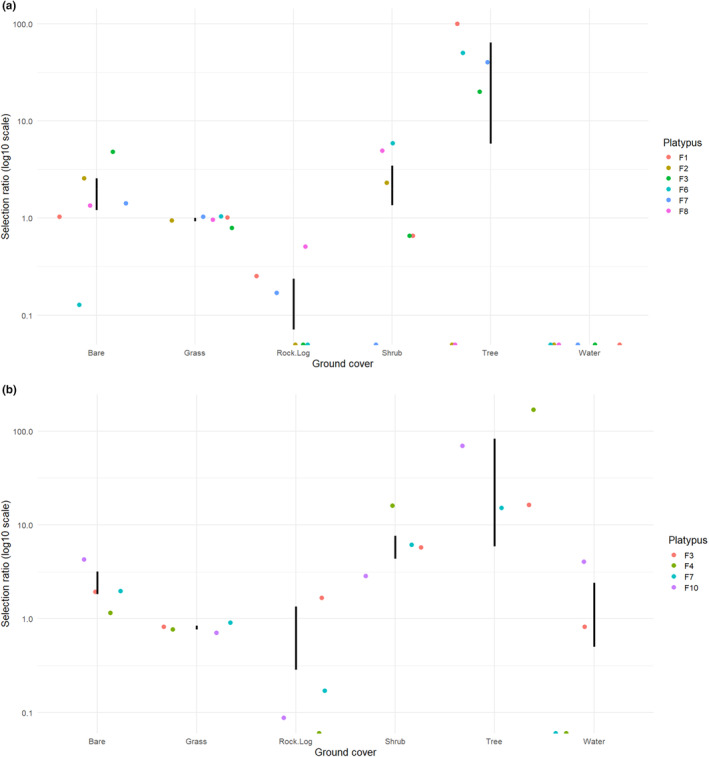
Manly section ratios for (a) nesting burrow and (b) resting burrow ground cover.

Female platypuses displayed strong habitat selection for ground cover within 5 m of their nesting burrows (*χ* ^2^L2 = 413.6, df = 30, *p* < .001, Table 4, Figure 5) with some variation among individual preferences (*χ* ^2^L1 = 202.4, df = 25.0, *p* < .001). Overall, the average level of selection across individual platypuses for nesting habitat deviated from available proportions (*χ* ^2^L2 − *χ* ^2^L1 = 211.2, df = 5, *p* < .001). The highest selectivity was for trees (wi = 29.165) and shrubs (wi = 2.404), with avoidance for Rock/Log (wi = 0.083). Female platypuses also displayed significant habitat preferences for ground cover within 5 m their resting burrows (*χ* ^2^L2 = 852.100, df = 20, *p* < .001), with some differential preferences between individuals (*χ* ^2^L1 360.921, df = 15, *p* < .001). Overall, the average level of selection for resting habitat across individuals deviated from available proportions (*χ* ^2^L2 − *χ* ^2^L1 = 492.078, df = 5.0, *p* < .001), with the highest selectivity for trees (wi = 44.560), shrubs (5.996), bare ground (wi = 2.499), and water (wi = 1.606).

**TABLE 4:** Summary of selection ratios for ground cover (5 × 5 m transects) above nesting and resting chambers.

Ground cover	Available	Used	Wi	SE	95% CI^a^
Nesting burrows					
Bare\Leaflitter	0.062	0.117	1.880	0.675	0.100–3.660
Grass\Rushes	0.865	0.832	0.962	0.038	0.862–1.063
Rock\Log	0.047	0.007	0.155	0.083	0–0.375
Shrub	0.012	0.029	2.404	1.053	0–5.183
Tree	<0.001	0.014	35.000	29.165	0–111.944
Water	0.013	0.000			
Resting burrows					
Bare\Leaflitter	0.062	0.156	2.500	0.671	0.730–4.269
Grass\Rushes	0.865	0.696	0.804	0.040	0.700–0.909
Rock\Log	0.047	0.039	0.815	0.530	0–2.213
Shrub	0.012	0.073	5.996	1.645	1.656–10.336
Tree	<0.001	0.018	44.560	38.640	0–146.501
Water	0.013	0.019	1.457	0.956	0–3.979

^a^ Negative values were altered to ‘0’ which is not logical for Manly Selective Index (Manly et al., 2007).

## 4 DISCUSSION

### 4.1 Nesting

Deriving accurate estimates of breeding requirements of wild platypuses is challenging given the species' cryptic nature, but essential to improve conservation outcomes in increasingly degraded freshwater ecosystems. To date, there have been no published studies that have successfully tracked multiple nesting females during the period of the breeding season that precedes egg hatching. This study provides valuable insights on breeding behaviour and preferences of platypuses and documents the temporal aspects of nest building for four female platypuses and presumptive egg‐laying dates for three of those females, with reference to a biological marker of egg yolk production.

Current estimates of the breeding period of wild platypuses range broadly, and are predominantly inferred from lactation detection in females and juvenile emergence (Bino et al., 2015; Grant et al., 2004; Hawke, Bino, & Kingsford, 2021) and limited observations of mating behaviour (Easton et al., 2008; Williams et al., 2012). Previous research in the study area estimated that breeding behaviour commenced in late September to early October (Hawke, Bino, Kingsford, Iervasi, et al., 2021). In this study, we further refine estimates, recording nesting behaviour (nest building and prolonged time spent in nesting burrow) over the month of September, suggesting the onset of breeding could be as early as late August to early September, given that the period between mating and retiring of the female to the nesting burrow varies from 13 to 23 days (Thomas et al., 2019).

Plasma triglyceride concentrations were higher than presumptive baseline values for 10 out of 11 females, providing support that observed nesting behaviour and time spent in burrows was associated with active folliculogenesis (maturation of the ovarian follicle/s) as in other egg laying species (O'Brien et al., 2016). F3 was the only female with plasma triglyceride concentrations below presumptive baseline values when bloods were taken on the 11 September. Tracking data however indicated that she was nesting from the 16 September, as she was using a single burrow for 14 consecutive days and was not located from this burrow for the first 9 days. It is possible that plasma triglyceride concentrations were below presumptive baseline values as the female had already laid eggs and commenced breeding. Tracking data indicated she had used a different burrow (F3B1) on the 13 September compared with the nesting burrow (F3B2), and therefore didn't meet our criteria until the 16 September. It was observed (in 2 instances) during radio tracking sessions if a tagged platypus was spotted in the water, they would go directly into resting burrows and stay there (confirmed with yagi on location). It is possible that F3 was already nesting prior to capture and tagging at F3B2 burrow, however, during the first tracking session she was disturbed by the operator of the radio tracker and entered a temporary burrow to hide (F3B1). As there is still much unknown about this species, it is recommended that further studies are conducted with nesting females earlier in the season, to capture them prior to nesting to limit cases like F3 where it is unknown when she began nesting due to conflicting data. Additional sampling of zoo‐based females to characterise plasma triglyceride concentrations during different reproductive states will provide further information on the utility of triglyceride as a biomarker of egg yolk production.

### 4.2 Habitat preferences

This study identified key habitat characteristics selected by female platypuses for nesting burrows. Female platypuses on the Snowy River displayed a strong selection for both shrubs and trees near their nesting and resting burrows. Resting burrows have been associated with tree density (Woon, 1995), and areas around trees or tree roots (Grant, 1983; Otley et al., 2000). Some studies suggest that platypuses do not have strict habitat requirements for resting burrows, which can be found in non‐earth structures such as vegetation (Otley et al., 2000; Serena et al., 1998). Unlike resting burrows, all nesting burrows were in consolidated earthen banks. Whilst platypuses also selected for bare ground, this was mostly representative of understory, as indicated by a preference for high canopy cover above the burrow chambers for nesting burrows. Results emphasise the importance of riparian vegetation along the banks of waterbodies to support platypus breeding. Debris from native vegetation such as *Lomandra* spp., and leaves and bark from Eucalyptus spp. are used in nests and are likely important in preventing eggs and young from desiccating (Hawkins & Battaglia, 2009; Thomas, Handasyde, et al., 2018). As burrow chambers are required to stay moist (Burrell, 1927; Thomas, Handasyde, et al., 2018), canopy cover from trees and shrubs may help in retaining soil moisture. Selection for burrows in the vicinity of trees and shrubs likely provides structural stability, as nesting burrows are complex structures (Thomas, Handasyde, et al., 2018), found to extend as much as 11.9 m within the banks (Temple‐Smith, 1973). This also prevents erosion, maintaining productive foraging areas, as platypuses prefer to forage in river cobbles and gravel rather than in silt (Grant et al., 2004), and organic matter increases macroinvertebrate populations and density (Boulton et al., 2014). By reducing erosion, bank height is also maintained, a key feature required for platypus nests to avoid drowning of young.

Platypuses also selected for high canopy cover above burrow entrances for both resting and nesting burrows compared with available habitat, a pattern previously identified elsewhere (Serena et al., 1998; Woon, 1995). Canopy likely provides cover during comings and goings from potential predators such as foxes, dogs, cats, and birds of prey. As nesting platypuses use the same burrow for up to 4 months before the young depart (Grant et al., 2004) these functionalities are particularly important. Trees and shrubs near the burrow entrances could also offer protection from predators (Grant, 2004) and stability for tunnel entrances (Temple‐Smith, 1973), suggestive of high shrub and tree presence along the water's edge near nesting burrows but not near resting burrow entrances. As resting burrow entrances have been found to be closer to the water or even underwater (Serena, 1994), trees and shrubs near entrances may not be a priority for platypus resting burrows.

Nesting burrows were also significantly higher above the water level and further from the water's edge than resting burrows, a possible adaptation to prevent burrows being inundated by rising water levels (Serena & Grant, 2017). Increased water levels because of a large release of water from the dam upstream may have resulted in a nest abandonment. Burrows on the Snowy River were found to be typically higher (1.34–3.14 m) and further away (6.40–13.4 m) in comparison with those identified elsewhere (1.1–1.4 m and 3.70–5.75 m) (Serena, 1994). This difference may be reflective of a behavioural adaptation by platypuses to the large flow volumes released from Jindabyne Dam on the Snowy River, with only one burrow noted to have likely been inundated (F6) by the large flushing flow release. Peak flows in previous years have been 13,000 ML/day (2017), 8617 ML/day (2018), 5000 ML/day (2019) and 4500 ML/day (2020), compared with 10,362 ML/day (2021) (Snowy Hydro, 2017, 2018, 2019, 2020, 2021). In the study area, which covered both natural habitats as well as disturbed pastureland, platypuses displayed varying habitat preferences, highlighting the range of habitat qualities. This range may have limited the need for adaptive plasticity, as optimal habitats were available. Choice of successful nesting burrow characteristics, such as height and distance from the water's edge, may also be a learned behaviour with lower younger females less likely to consider larger flow events. Management of the timing of large flow releases from dams in Australia's regulated rivers should consider the possible impact on nesting platypuses, particularly in rivers where regulation has disassociated the timing of release with the natural flow regime. This provides an important avenue for conservation management of regulated rivers to ensure that platypuses are able to maintain recruitment.

In managed settings, breeding females have been observed to display competitive behaviour (Thomas, Parrott, et al., 2018). While competition for nesting material and burrow locations is possible, we observed females nesting in proximity, with two females found nesting within 4 m of each other, and all identified nesting burrows located within a 405 m section of the river. Whether the observed spatial clumping is a result of clumped resources or a preference for colonial nesting remains unknown and should be a future research direction. Determining whether this clumping is a preference or results from limited options due to river and riparian management is key, as high densities of nesting burrows and subsequent juvenile emergence may increase susceptibility to threats, possibly reducing breeding success, as suggested in a recent study in the area (Hawke, Bino, & Kingsford, 2021).

### 4.3 Implications for conservation

Clearing of riparian vegetation and livestock impacts have both been attributed as the main causes of freshwater degradation in platypus habitats in Thredbo (Goldney, 1998), Eden (Lunney et al., 1998) and Bellinger catchment (Lunney et al., 2004) resulting in fragmentation of platypus populations, threatening the long‐term viability of populations (Bino et al., 2021). On two occasions, platypus burrows had caved in from cattle grazing along the banks, possibly leading to nest abandonment, trampling of young or exposure to predators. This study highlights the importance of riparian vegetation for breeding of platypuses with direct relevance to the conservation of a species in decline. Australia has one of the highest land clearing rates in the world (Bradshaw, 2012; Evans, 2016; Reside et al., 2017). Continued land clearing and degradation of both terrestrial and freshwater habitats across the range of platypuses threatens the species with further declines (Hawke et al., 2020).

Within the study area, encompassing both intact riparian vegetation and livestock‐degraded areas, female platypuses displayed a high degree of selection for their nesting burrows. In line, conservation efforts should be prioritised to conserve and improve riparian vegetation in freshwater systems to increase habitat which can support breeding. Restoration efforts should be made to bare banks that are more susceptible to erosion which increases sedimentation and decreases water quality. A minimum 20 m buffer to prevent livestock access along these essential bank habitats should also be a focus to maintain riparian vegetation, bank stability and prevent trampling of burrows. Management teams for regulated rivers need to consider impacts of large water releases and transfers during peak breeding times and seek out alternatives to prevent drowning of young platypuses.

Breeding programs of platypus have had limited success to date. Since the first zoo‐bred platypus in 1943, breeding has only been successfully facilitated in six pairs, some of which were successful on multiple occasions (Thomas, Parrott, et al., 2018). This limited success has been largely attributed to deficiencies in knowledge of the species' specific breeding requirements. However, recent studies have made strides in identifying key features of nesting burrows and the types of nest vegetation material preferred by captive platypuses (Thomas, Handasyde, et al., 2018). Our findings suggest that ex situ breeding programs should provide high earth banks with shrubs and cover at the water's edge to best replicate wild nesting habitats and enhance reproductive success rates. This study is one of the first to track multiple nesting females and determine habitat selection; however, it is important that this work is replicated and expanded, particularly in different ranges of the platypus, to ensure that the resource selection observed is not unique to this population on the Snowy River.

Further research may also be required to assess predation by introduced rodents. Black rats (*Rattus rattus*) and mice (*Mus musculus*), both invasive species to Australia, were observed visiting nesting burrows on several occasions. While it was not apparent that any had managed to predate on platypus young or eggs, their presence is suggestive of a potential predation risk for unguarded altricial platypus young, as black rats are known to predate on bird eggs and chicks (Brown, 1997). Platypuses are known to pug nesting burrow tunnels (Burrell, 1927) as a possible defensive measure and climatic control for eggs and young, although not all nesting burrows are found to have pugs (Thomas, Handasyde, et al., 2018). Whether rats can detect young and dig through such pugs remain unknown, but findings highlight the need for further research. Overall, efforts to unravel key knowledge gaps of the species' breeding biology are of high priority to support ongoing conservation efforts of this cryptic animal in decline.

## AUTHOR CONTRIBUTIONS

**Joseph Crane:** Formal analysis (lead); writing – original draft (lead); writing – review and editing (lead). **Gilad Bino:** Conceptualization (lead); formal analysis (equal); supervision (lead); writing – original draft (equal); writing – review and editing (equal). **Neil R. Jordan:** Formal analysis (equal); supervision (equal); writing – original draft (equal); writing – review and editing (equal). **Tahneal Hawke:** Formal analysis (equal); supervision (equal); writing – original draft (equal); writing – review and editing (equal). **Justine K. O'Brien:** Formal analysis (equal); writing – original draft (equal); writing – review and editing (equal).

## FUNDING INFORMATION

This study was funded by the Taronga Conservation Society Australia and University of New South Wales.

## CONFLICT OF INTEREST STATEMENT

The authors declare that they have no conflicts of interest.

## Supporting information


Appendices S1–S3

## Data Availability

The data and R code that support the findings of this study are available in Dryad https://datadryad.org/stash/share/dPNf2IVaG00drgUIn0G73EU‐TJKWDiAU5sYc1toKEhI.
