# Supplementary material for: From banks to burrows: Habitat preferences and nesting behaviours of platypuses in the Snowy River
Source: Ecol Evol. 2024 Dec 24;14(12):e70347. doi: 10.1002/ece3.70347 (PMC11666990; doi:10.1002/ece3.70347)

# Appendices

## Appendix S1: Platypus caught during surveys (10/9/2021 – 15/9/2021, 12/10/2021 -15/10/2021), their location, sex (Female/Male), age (Adult/Sub-Adult/Juvenile), Tail Volume Index (TVI), weight [kg], and morphometrics [mm].

| ID | Location | Sex | Age | TVI | Weight | Bill Length shield/no shield | Bill width | Total body length | Tail length | Tail width |
| --- | --- | --- | --- | --- | --- | --- | --- | --- | --- | --- |
| F1 | W | F | A | 1 | 1.00 | 65/50 | 45 | 440 | 105 | 70 |
| F2 | G | F | A | 2 | 1.00 | 68/50 | 44 | 440 | 122 | 71 |
| F3 | G | F | A | 1 | 0.91 | 64/47 | 42 | 410 | 112 | 71 |
| F4 | W | F | A | 2 | 0.84 | 63/45 | 41 | 408 | 128 | 70 |
| F5 | W | F | A | 3 | 0.88 | 63/46 | 40 | 448 | 125 | 70 |
| F6 | G | F | A | 3 | 0.94 | 64/48 | 43 | 441 | 135 | 71 |
| F7 | G | F | A | 3 | 0.79 | 63/47 | 38 | 425 | 132 | 70 |
| F8 | G | F | A | 2 | 0.98 | 65/48 | 44 | 430 | 133n | 77 |
| F9 | W | F | A | 4 | 0.77 | 72/54 | 47 | 438 | 125 | 60 |
| F10 | W | F | A | 5 | 0.81 | 64/59 | 42 | 430 | 115 | 70 |
| F11 | W | F | A | 4 | 0.82 | 67/47 | 43 | 445 | 125 | 67 |
| Male | W | M | A | 5 | 1.14 | 70/53 | 46 | 475 | 120 | 55 |
| Male | H | M | A | 5 | 1.20 | 70/53 | 50 | 495 | 140 | 65 |
| Male | W | M | A | 5 | 1.18 | 77/57 | 52 | 494 | 147 | 63 |
| Male | W | M | A | 4 | 1.60 | 82/58 | 51 | 534 | 160 | 80 |
| Male | W | M | A | 3 | 1.39 | 74/60 | 52 | 515 | 150 | 80 |
| Male | H | M | A | 3 | 1.18 | 76/57 | 49 | 475 | 138 | 65 |

## Appendix S2: Platypus survey locations, number of nights surveyed (10/9/2021 – 15/9/2021, 12/10/2021 -15/10/2021), number of platypuses captured and their sex.

| Location | Nights | Captures (recaptures) | Males | Females |
| --- | --- | --- | --- | --- |
| W | 5 | 10 (1) | 4 | 6 |
| G | 2 | 5(0) | 0 | 5 |
| H | 3 | 2(1) | 2 | 0 |
| Total | 10 | 17(2) | 6 | 11 |

## Appendix S3: Locations of the ten tagged female platypuses during the tracking period 13/9/2021 – 25/11/2021


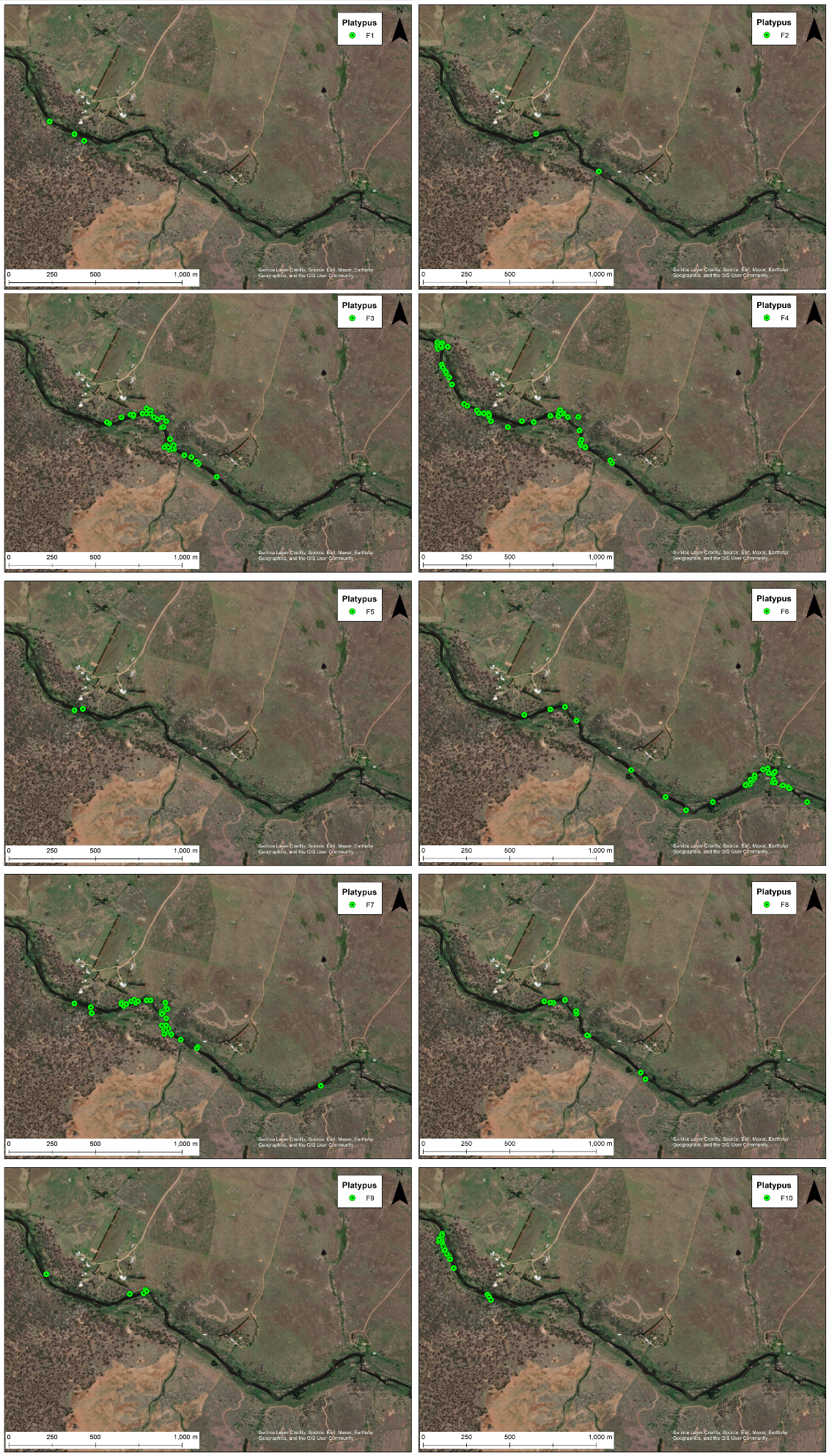

Supplement: Supplementary file 1 — Appendices S1–S3 [file ECE3-14-e70347-s001.docx]
